# Clinical outcomes of levosimendan versus dobutamine in patients with acute decompensated heart failure with reduced ejection fraction and impaired renal function

**DOI:** 10.1016/j.ihj.2021.02.010

**Published:** 2021-02-17

**Authors:** Basil John, Mariya Babu, Sandramol shaji, Suja Abraham, Jabir Abdullakutty

**Affiliations:** a Nirmala College of Pharmacy, Muvattupuzha, Ernakulam District, Kerala, India; b Department of Pharmacy Practice, Nirmala College of Pharmacy, Muvattupuzha, Ernakulam District, Kerala, India; c Lisie Hospital in Cochin, Kerala, India

**Keywords:** Levosimendan, Dobutamine, Acute decompensated heart failure, Cardiorenal syndrome

## Abstract

To assess the clinical outcomes of levosimendan and dobutamine in patients with acute decompensated heart failure with reduced ejection fraction and impaired renal function in Indian scenario. Cardiac, renal, electrolytes and hepatic parameters as well as the clinical outcomes were assessed. Levosimendan and dobutamine improved ejection fraction significantly. Levosimendan in comparison to dobutamine, increased cardiac output (0.76 vs. −0.38 at 48 h, 1.15 vs. −0.31 day 7, -2.02 vs. −1.51 day 30), cardiac index (0.89 vs.-0.13 at 48 h, 1.16 vs. −0.07 at day 7 and 1.05 vs. −0.25 at day 30) and eGFR (−1.4 vs. −0.75 at day 30) significantly. Levosimendan reduced ICU stay (*p* = 0.038) significantly whereas dobutamine decreased the hospital stay duration (*p* = 0.015). There was no major difference in re-hospitalization and mortality between groups. Ventricular tachyarrhythmia was the main adverse event noted in Levosimendan arm. Levosimendan showed improved cardiac as well as renal outcomes within a month when compared to dobutamine and it is the first study to determine the renal parameters of Levosimendan in an Indian setting.

## List of abbreviations

ADHF: Acute decompensated heart failure
GFR: glomerular filtration rate
BP: blood pressure
HR: heart rate
EF: ejection fraction
SV: stroke volume
CO: cardiac output
CI: cardiac index
LVESD: left ventricular end systolic diameter
S.cr: serum creatinine

## 1 Introduction

Cardio-renal syndrome is a major complication of acute decompensated heart failure, which is a life-threatening state defined by worsening fatigue, dyspnoea or oedema that result from deteriorating heart function that results in reduced cardiac output (CO) and glomerular filtration rate (GFR).^1^ The development of Inotropes provides clinical and symptomatic improvement for patients with reduced CO and compromised vital organ functions.^2^

Levosimendan is an inotrope found to increase myocardial contractility through calcium dependent binding to cardiac troponin C and benefits patient with cardiorenal syndrome by increase in renal blood flow, vasodilatation, and possess anti-inflammatory effects against tubular injury.^3^^,^^4^ Incidence of atrial fibrillation, hypokalemia, and headache during the in-drug administration is higher in levosimendan when compared with dobutamine.^5^ There were studies that focus on hemodynamic and clinical effects of Levosimendan and Dobutamine but very limited studies exist on the renal effects. Our study is probably the only study in India which compares the renal outcomes of these inotropes in the management of ADHF. Published literature still puts up a controversy about their comparative effectiveness. So, we aim to evaluate the cardiac, renal and clinical efficacy of these agents in ADHF patients with renal impairment.

## 2 Materials and methods

The study was a prospective, comparative and open label randomized controlled study, conducted in a tertiary cardiac care hospital in Kochi from November 2018 to April 2019. Prior to the initiation of the study, the protocol was reviewed and approved by the institutional ethics committee and Informed consent was obtained from each study participant before their enrolment into the study and they were assured the confidentiality of the data collected.

### 2.1 Sample size calculation

T tests - Means: Difference between two independent means (two groups).

Analysis: A priori: Compute required sample size.

Input: Tail(s) = Two.

Effect size *d* = 0.9643759

α err prob = 0.05.

Power (1-β err prob) = 0.8.

Output:

Sample size group 1 = 15.

Sample size group 2 = 15.

Total sample size = 30.

A total of 58 participants were randomised in 1:1 ratio to obtain 22 in levosimendan and 36 in dobutamine group. Etiology of ADHF includes MI, arrhythmia, valvular dysfunction, hypertensive emergency, renal as well as hepatic dysfunction and the patients were treated with diuretics, vasodilators, inotropes, vasopressors and thromboembolic prophylaxis according to ESC guidelines. On CCU admission, the patients were screened to assess their eligibility-

● Signed Informed consent from patients, Acute decompensated Heart failure patients with age ≥18 years, left ventricular ejection fraction (EF) ≤30%, eGFR between 30 and 60 ml/min/1.73 m^2^ (MDRD).
● Patients with SB*P* < 90 mmHg, heart rate (HR) > 12Obpm, serum potassium <3.5 mmol/l, Pulmonary embolism, Hypertrophic cardiomyopathy were excluded from the study.
  • For randomization, Patients were administered Levosimendan on every even day as a loading dose of 12mcg/kg over 10 min followed by a 0.1mcg/kg/min infusion, administered for 24 h and Dobutamine on odd days as infusion of 5mcg/kg/min (maximum −20mcg/kg/min).
  • The cardiac, hepatic and renal parameters were followed up after 48hrs, 7th day or before discharge whichever came earlier and on the 30th day.
  • Cardiac parameters were assessed by modified Simpson’s method and eGFR was calculated using Modification of Diet in Renal Disease (MDRD) equation:

186 x (Creatinine/88.4)^−1.154^ x (Age)^−0.203^ x (0.742 if female) x (1.210 if black)

### 2.2 Statistical analysis

For statistical analysis, SPSS version 24 was used. All the p values were two-tailed and a significance level of 5% was used. Data storage was done using Microsoft excel.

## 3 Result

To compare the clinical effects of Levosimendan and Dobutamine, in ADHF patients with impaired renal function, a total of 58 patients were enrolled into the study.

### 3.1 Baseline demographics and clinical characteristics

Table 1 demonstrates the baseline demographics and clinical characteristics between the study groups. The study population belongs to an age group of 63.47 ± 12.23 years. In Levosimendan group out of 22 patients, 5 were females and 17 were males whereas in Dobutamine out of 36, there are 8 females and 28 males. Therefore, a male predominance was noted in both the groups.

**Table 1:** Baseline demographics and clinical characteristic.

Parameters	Levosimendan	Dobutamine	*p* value
Age			
Mean ± SD	60.64 ± 11.00	65.19 ± 12.77	0.171
Weight	63.55 ± 12.23	63.47 ± 12.64	0.983
Height	164.36 ± 8.72	165.82 ± 8.76	0.548
BP Systolic	127.82 ± 21.33	129.75 ± 21.13	0.738
BP Diastolic	79.95 ± 16.93	75.25 ± 13.15	0.242
Heart Rate	90.05 ± 14.81	86.31 ± 15.30	0.365
Ejection Fraction	25.20 ± 4.52	25.86 ± 4.18	0.575
Stroke Volume	36.29 ± 14.98	42.87 ± 18.79	0.171
Cardiac output	3.16 ± 0.99	3.45 ± 1.30	0.389
Cardiac Index	1.80 ± 0.60	1.95 ± 0.78	0.436
Left Atrial Volume	50.03 ± 32.48	47.36 ± 17.53	0.689
LVESD	4.41 ± 1.59	4.24 ± 1.61	0.693
LVEDD	5.35 ± 1.03	5.29 ± 0.82	0.798
LVESV	96.25 ± 50.37	98.32 ± 54.54	0.886
LVEDV	134.82 ± 57.36	140.71 ± 61.73	0.719
Urea	51.60 ± 27.56	56.34 ± 41.87	0.690
Creatinine	3.25 ± 2.19	2.52 ± 0.58	0.129
eGFR	37.60 ± 13.59	41.71 ± 14.49	0.288
Uric acid	10.77 ± 4.692	10.97 ± 7.27	0.606
Sodium	136.95 ± 4.80	136.72 ± 6.04	0.879
Potassium	4.70 ± 0.51	4.38 ± 0.57	0.038
Bilirubin	0.91 ± 0.84	0.96 ± 0.81	0.820
SGPT	45.18 ± 40.67	71.94 ± 129.39	0.352
Hemoglobin	15.45 ± 1.85	14.89 ± 2.46	0.159

BP- blood pressure, LVESD-left ventricular end systolic diameter, LVEDD -left ventricular end diastolic diameter, LVESV- left ventricular end systolic volume, LVEDV- left ventricular end diastolic volume, GFR-glomerular filtration rate, SGPT-serum glutamate pyruvate transaminase.

In Levosimendan group, the major etiologies were found to be diabetes (68.18%), hypertension (36.36%) and coronary heart disease (36.36%). Dobutamine group had a similar etiology; diabetes (66.67%), hypertension (69.44%) and coronary heart disease (25%). Both, the groups had comparable contributing factors except for hypertension (*p* = 0.01).

### 3.2 Assessment of cardiac parameters

Cardiac parameters monitored were blood pressure (BP), HR, EF, CO, stroke volume (SV), cardiac index (CI), left atrial volume, left ventricular end systolic diameter (LVESD), left ventricular end diastolic diameter, left ventricular end systolic volume and left ventricular end diastolic volume. A statistical significance was observed only for parameters like EF, CO, CI and LVESD.

Levosimendan decreased HR from baseline to 48 h (*p* = 0.035) and to 7th day (*p* = 0.030). EF in the Levosimendan arm, improved from baseline to 48 h, 7th day and 30th day and was statistically significant (all *p* = 0.000). EF rise from baseline to 48 h in dobutamine arm was statistically significant (*p* = 0.02) whereas HR was found to decrease (*p* = 0.06). When compared to dobutamine, Levosimendan improved EF significantly at 48 h (*p* = 0.03), 7th day (*p* = 0.003) and 30th day (*p* = 0.008).

There was no significant difference in CO or CI within the group either in Levosimendan or dobutamine arm. However, levosimendan showed clinically evident improvement in CO and CI. Dobutamine had an initial improvement in CO and CI but showed a decline after 7th day whereas the effect persisted in levosimendan arm. This could have led to a statical difference in levosimendan arm in comparison to dobutamine (as in Table 2).

**Table 2:** Comparison of Cardiac output, LVESD, creatinine between Levosimendan and Dobutamine.

Parameter	Time period	Drug	Mean difference ± Std. Deviation	*p* value
Cardiac output	*48 h*	*Levosimendan*	0.76 ± 1.302	**0.004**
		*Dobutamine*	−0.38 ± 1.370	
	*7*^*th*^*day*	*Levosimendan*	1.15 ± 1.306	**0.001**
		*Dobutamine*	−0.31 ± 1.168	
	*30*th *day*	*Levosimendan*	2.02 ± 0.810	**0.028**
		*Dobutamine*	−1.51 ± 0.954	
Left ventricular end systolic diameter	*48 h*	*Levosimendan*	0.25 ± 0.872	0.061
		*Dobutamine*	−0.47 ± 1.605	
	*7*^*th*^*day*	*Levosimendan*	0.46 ± 1.325	**0.037**
		*Dobutamine*	−0.51 ± 1.447	
	*30*th *day*	*Levosimendan*	0.26 ± 0.984	0.480
		*Dobutamine*	−0.52 ± 1.263	
Serum creatinine	*48 h*	*Levosimendan*	−0.4511 ± 1.28363	0.918
		*Dobutamine*	−0.4892 ± 1.11333	
	*7*th *day*	*Levosimendan*	−0.2668 ± 1.33321	0.137
		*Dobutamine*	0.3009 ± 1.05909	
	*30*th *day*	*Levosimendan*	−1.4126 ± 1.93357	**0.001**
		*Dobutamine*	0.7588 ± 1.59993	

**Fig. 1:**
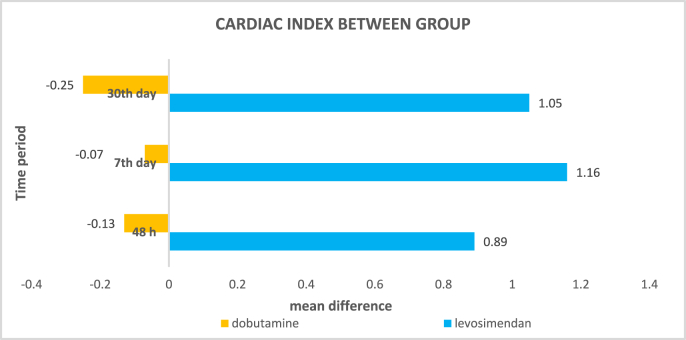
Cardiac Index between levosimendan and dobutamine.

Levosimendan showed statistically significant increase in CI at all-time points in comparison to Dobutamine as in Fig. 1 (*p* = 0.001 at 48 h and *p* = 0.000 for 7th and 30th day).

Reduction in LVESD improves prognosis of the patients. On 7th day dobutamine showed a greater decrease in LVESD compared to Levosimendan in Table 2.

For all the parameters other than the above, there was no significant improvement either within the group or between them.

### 3.3 Assessment of non-cardiac parameters

Non-cardiac parameters like renal function, electrolytes and hepatic function were assessed.

As shown in Table .2, Levosimendan reduced serum creatinine (S.c*r*) at all time-points but a significant difference was observed at 30th day compared to the baseline (*p* = 0.028). eGFR showed significant improvement from baseline to 48 h (*p* = 0.003) and to 7th day (*p* = 0.03).

**Fig. 2:**
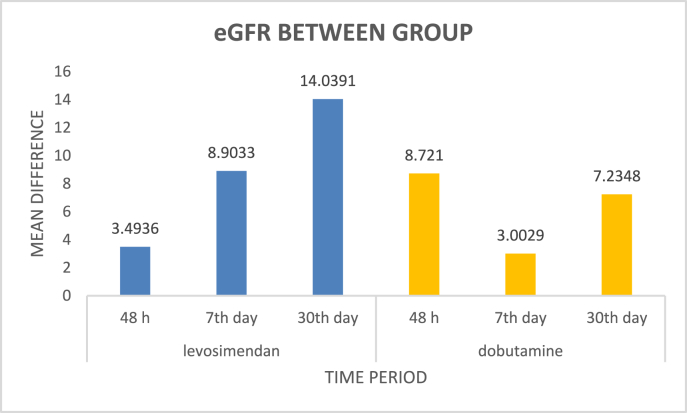
Difference in eGFR between Levosimendan and Dobutamine.

Dobutamine showed an initial decline in S. c*r* followed by a significant rise at day 30 from baseline (*p* = 0.028). However, increase in eGFR was noted on day 2 and 7 compared to baseline (*p* = 0.003 and 0.03 respectively). Levosimendan reduced S. c*r* significantly at day 30 leading to a rise in eGFR (*p* = 0.001) compared to dobutamine as illustrated in Fig. 2.

Uric acid was decreased significantly by Levosimendan at day 7 compared to baseline (*p* = 0.041) and by Dobutamine at day 7 and day 30 (*p* = 0.05 and 0.032 respectively). Levosimendan was found to decrease potassium at 48 h and at day 7 (*p* = 0.00, 0.009 respectively). Dobutamine showed decline in potassium levels after 48 h post-infusion (*p* = 0.006). There was no significant difference in other parameters, either by Levosimendan or by dobutamine.

### 3.4 Assessment of the clinical outcomes

The clinical outcomes assessed were length of ICU and total hospital stay, rehospitalisation, and mortality.

Levosimendan reduced ICU stay significantly (*p* = 0.038), whereas dobutamine decreased the hospital stay duration (*p* = 0.015). The longest hospital stay in levosimendan group was 17 days due to recurrent ventricular tachycardia experienced by a patient and this could have influenced the overall mean of hospital stay in levosimendan arm. Readmissions of patients within one month after discharge showed no statistical difference (*p* = 0.387) between groups.

Three patients in levosimendan group showed ventricular tachycardia and one patient experienced hypotension. In Dobutamine group, one patient died due to severe bradycardia.

Mortality within 7 days of drug infusion (In-hospital Mortality) and within 30 days (outside-hospital setting) was assessed separately. A total of 3 deaths were observed in Levosimendan arm and 11 in Dobutamine. The rate of in-hospital mortality in levosimendan is much lower (5%) than the dobutamine arm (22%) (*p* = 0.071). There was no statistical difference in the 30-day mortality (excluding the in-hospital deaths) and overall mortality among the groups (*p* = 0.921, *p* = 0.123 respectively).

## 4 Discussion

In our study, HR was found to be lower in patients treated with levosimendan and Dobutamine but there was no significant difference between the groups. In LIDO trial both the drugs increased the HR to similar extent.^6^ We observed a significant increase in EF in both the groups but it was more profound in levosimendan group. This is identical to the results obtained from study conducted by D. Hamza et al.^7^

Levosimendan improved CO better than Dobutamine at 48 h, day 7 and day 30. In another study, it was found that the improvement provided by Levosimendan was statistically greater.^8^ In our study, Levosimendan was found to improve CI significantly at all-time points in comparison to Dobutamine. This is almost similar to the values obtained in the study done by A. Julian.^9^ A significant decrease in LVESD was shown by dobutamine after 7th day compared to levosimendan. In another study, LVESD reduced with Levosimendan but no change was seen in Dobutamine group.^10^

Levosimendan decreased S. c*r* at day 30 and increased eGFR significantly in comparison to Dobutamine. In a study it was found that Levosimendan had a short-term effect on S. c*r* on day 1 and 3 but no profound effect was noticed on day 10 compared to standard therapy.^11^

In our study Levosimendan decreased the length of ICU stay significantly compared to Dobutamine as was in the findings by Kandaswamy A *et al*^12^ We observed a significant increase in hospital stay duration in Levosimendan arm compared to Dobutamine which contrast the results obtained by MaderiaM*et al*.^13^ In a trial conducted by Farmakis D *et al* as well as in REVIVE, Levosimendan showed no decrease in the rates of rehospitalisation compared to the control.^14^^,^^15^ We obtained an insignificant difference in mortality within hospital or at 30 days between Levosimendan and Dobutamine which is similar to result in SURVIVE.^5^

## 5 Conclusion

Our aim is to assess the clinical outcomes of levosimendan and dobutamine in patients with ADHF and impaired renal function in the Indian scenario. Levosimendan was found to have a profound action on EF, CO, CI and eGFR. Dobutamine improved EF and eGFR from baseline but these effects were short-termed. There was no statistical difference in mortality between the groups but was clinically significant with 11 deaths in Dobutamine arm. Therefore, Levosimendan has improved cardiac as well as renal outcomes within a month when compared to dobutamine.

## Funding

This research did not receive any specific grant from funding agencies in the public, commercial, or not-for-profit sectors.

## Declaration of competing interest

The authors declare no conflict of interest.
